# Co-infection of tuberculosis and parasitic diseases in humans: a systematic review

**DOI:** 10.1186/1756-3305-6-79

**Published:** 2013-03-22

**Authors:** Xin-Xu Li, Xiao-Nong Zhou

**Affiliations:** 1 National Institute of Parasitic Diseases, Chinese Center for Disease Control and Prevention, Key Laboratory of Parasite and Vector Biology, Ministry of Health, WHO Collaborating Centre for Malaria, Schistosomiasis and Filariasis, Shanghai, 200025, P. R. China; 2 National Center for Tuberculosis Control and Prevention, Chinese Center for Disease Control and Prevention, Beijing, 102206, P. R. China

**Keywords:** Tuberculosis, Parasitic diseases, Co-infection

## Abstract

Co-infection of tuberculosis and parasitic diseases in humans is an important public problem in co-endemic areas in developing countries. However, there is a paucity of studies on co-infection and even fewer reviews. This review examines 44 appropriate papers by PRISMA from 289 papers searched in PubMed via the NCBI Entrez system (no grey literature) up to December 2012 in order to analyze the factors that influence epidemic and host’s immunity of co-infection. The limited evidence in this review indicates that most common parasite species are concurrent with *Mycobacterium tuberculosis* in multiple organs; socio-demographics such as gender and age, special populations with susceptibility such as renal transplant recipients, patients on maintenance haemodialysis, HIV positive patients and migrants, and living in or coming from co-endemic areas are all likely to have an impact on co-infection. Pulmonary tuberculosis and parasitic diseases were shown to be risk factors for each other. Co-infection may significantly inhibit the host’s immune system, increase antibacterial therapy intolerance and be detrimental to the prognosis of the disease; in addition, infection with parasitic diseases can alter the protective immune response to Bacillus Calmette-Guerin vaccination against *Mycobacterium tuberculosis*.

## Introduction

### Rationale

Both tuberculosis (TB) and parasitic diseases in humans are infectious diseases that exhibit an extensive distribution, causing serious harm to humans. The World Health Organization Special Programme for Research and Training in Tropical Diseases (WHO TDR) provided the TDR disease portfolio in 1999 to deal with the deterioration in the health situation, including leprosy, TB and eight kinds of parasitic diseases, such as malaria, schistosomiasis, etc. [1]. WHO estimated that there was about one third of the global population infected by TB, and in 2010, there were an estimated 8.8 million incident cases of TB globally, mostly occurring in Asia (59%) and Africa (26%) [2]. Meanwhile, in 2009 WHO also reported that there were an estimated 225 million malaria cases, mainly distributed in Africa (78%), South-East Asia (15%) and the Eastern Mediterranean (5%) [3]. In 2012, there were an estimated 436 million people at risk of *Schistosomiasis haematobium* infection in Sub-Saharan Africa, of which 112 million were infected, with an estimated 393 million people at risk of *Schistosomiasis mansoni* infection, of which 54 million were infected [4]; an estimated 120 million people in tropical and subtropical areas of the world were infected with lymphatic filariasis in 2009 [5]. These figures suggest that there is an overlap of endemic regions between TB and parasitic disease, which may lead to co-infection of these diseases in the population.

The earliest report we found was from 1945 and interpreted how to treat a pulmonary TB (PTB) case running concurrently with malaria [6]. A report from 1946 described co-existence of TB with hookworm [7]. The co-infection of TB and parasitic diseases have been reported in many studies for almost the past 70 years, although great achievements have been gained in the fields of TB and parasitic disease control and prevention respectively [2-5]. Up to 2012, some cases of co-infection between TB and parasitic diseases were reported around the world [8-29], and some epidemiological surveys of co-infection in hospitals or communities were carried out [30-34]. Some of these studies showed that the immune response was modified in the co-infection situation [35-51].

Inevitably, co-infection would increase the complexity of control and prevention on TB and parasitic diseases. The current systematic reviews on the co-infection of TB and parasitic diseases help to clarify the complexity of co-infections; however, there are only a few systematic reviews on co-infection. We only found that Enwere *et al.*[35] reviewed the host response of co-infection between TB and malaria and 4 reviews focused on the influence of chronic helminth infections on immunity against TB [44,45,47,48].

### Objectives

This paper reviewed studies globally over the past 70 years on the co-infection of TB and parasitic diseases using Preferred Reporting Items for Systematic Reviews and Meta-Analyses (PRISMA) guidelines [52], in order to learn more about which parasites are concurrent with TB, the epidemiological situation regarding co-infection, and the human immune function affected by co-infection.

## Methods

### Protocol and registration

We did not register the protocol for this review.

### Eligibility criteria

Published articles were included if they involved case reports of co-infection with TB and any parasitic diseases in human participants, or epidemiological surveys of co-infection in populations, or clinical or laboratory research on the immune responses during co-infection, were eligible for inclusion in the systematic review. Journal articles published with full text or abstracts in English before 2013 were eligible for inclusion.

### Information sources

We mainly searched PubMed via the NCBI Entrez system (any date to December, 2012) (http://www.ncbi.nlm.nih.gov) for studies on the association between TB and parasitic diseases. We also searched bibliographies of identified reports, including previous reviews, for additional references.

### Search

The search was limited to studies of human beings or animal models and no language limits were applied. The terms that were used as MeSH terms or Direct keywords for the search and our search strategy are described in Table 1. All titles, abstracts and full texts from each of the searches were examined and reviewed.

**Table 1:** Search strategy and terms used to identify studies on co-infection between TB and common parasitic diseases

**Term**	1. tuberculosis, 2. parasite, 3. helminth, 4. amoebiasis, 5. leishmaniasis, 6. trypanosomiasis, 7. giardiasis, 8. trichomoniasis, 9. malaria, 10. toxoplasmosis, 11. cryptosporidiosis, 12. clonorchiasis, 13. opisthorchiasis, 14. fasciolopsiasis, 15. fascioliasis, 16. paragonimiasis, 17. schistosomiasis, 18. sparganosis, 19. echinococcosis, 20. ascariasis, 21. trichuriasis, 22. enterobiasis, 23. hookworm, 24. strongyloidiasis, 25. trichinellosis, 26. filariasis, 27. dracunculiasis
**Strategy**	1 and (2 or 3 or 4 or 5 or 6 or 7 or 8 or 9 or 10 or 11 or 12 or 13 or 14 or 15 or 16 or 17 or 18 or 19 or 20 or 21 or 22 or 23 or 24 or 25 or 26 or 27)

### Study selection

**Figure 1:**
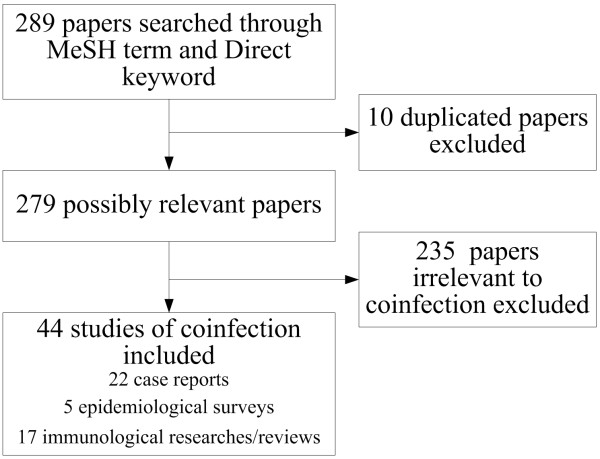
Study selection information regarding co-infection between TB and parasitic diseases.

The study selection process is illustrated in Figure 1.

Papers that were not co-infection studies, or were not for humans, or were published in non-English language without an abstract in English, were excluded. Eligible papers were tabulated.

### Data collection process

Two reviewers extracted data from each eligible study independently, and differences were resolved by discussion with a third. Extracted data was tabulated on the basis of data items. No formal meta-analysis was carried out and analysis to investigate statistical heterogeneity or publication bias was not performed because most of the studies were case reports and immunological research, and there were very few epidemiological surveys.

### Data items

Data extracted included country, year, co-infection of diseases, sex, age, human immunodeficiency virus (HIV) test and medical history for case reports. For epidemiological surveys, the detailed information included was country, year, survey site, number of population screened, number of people with PTB, number of people with parasitic disease, number of people co-infected and prevalence. For clinical or laboratory research on immunity during co-infection, we collected detailed information on country, year, co-infection of disease, subject and conclusions.

### Summary measures

Risk ratios (RR) and their 95% confidence intervals (CI) were calculated using the Statistical Analysis System (SAS 9.2; SAS Institute Inc., Cary, NC, USA) through Chi square test. All *P*-values of <0.05 were taken as statistically significant.

## Results

Two hundred and eighty nine papers were retrieved from the search of published work, of which, 10 were excluded because of duplication and 235 were irrelevant to co-infection with TB and parasitic diseases. Finally, 44 studies of co-infection were included in the analysis, of which, 22 were case reports, 5 were epidemiological surveys and 17 were immunological research/reviews (Figure). There was no grey literature included.

Table 2 summarizes the case reports of co-infection. Twenty-four cases were reported in 22 studies from 13 countries during 1984 to 2012, in which 7 cases were from India. Fourteen studies investigated co-infection with PTB and parasites diseases and 8 studies involved the extrapulmonary TB, such as renal TB, lymph node TB, abdominal TB, TB in liver, tuberculous lymphadenitis and TB verrucosa cutis. The youngest case reported was 34 days old and the eldest case was 82 years old, and the median age of all cases reported was 36 years old. Fifteen of a total 24 cases were male. Ten patients with different clinical conditions co-infected with TB and leishmaniasis were reported, of which, visceral leishmaniasis was the highest. There were also 6 cases co-infected with TB and hydatid disease. In addition, parasitic diseases accompanied by TB included trichomoniasis, malaria, toxoplasmosis, toxocariasis, schistosomiasis, strongyloidiasis and filarial elephantiasis in different organs. Fourteen of a total of 24 cases reported had been tested for HIV test and 4 were positive.

**Table 2:** Case reports of co-infection between TB and parasitic diseases

**Country**	**Year of report**	**Tuberculosis**	**Parasitic disease**	**Sex**	**Age**	**HIV test**	**Supplement**	**No. of reference**
Spain	1996	Renal TB	Visceral leishmaniasis	Male	44 years	Negative	Haemodialysis for 19 months	[8]
Brazil	2001	PTB	Visceral leishmaniasis	Male	29 years	Negative	*Pneumocystis carinii* positive	[9]
Turkey	2003	PTB	Visceral leishmaniasis	Male	39 years	Negative	Renal transplantation in 2001; Anti-HCV and HCV RNA positive	[10]
India	2005	PTB	Visceral leishmaniasis	Male	37 years	Positive	Tuberculoma of brain	[11]
India	2006	PTB	Visceral leishmaniasis	Female	40 years	Positive		[12]
India	2006	PTB	Visceral leishmaniasis	Male	40 years	Positive		[12]
Colombia	1996	PTB	Mucocutaneous leishmaniasis	Male	50 years	Negative		[13]
France	2003	PTB	Cutaneous leishmaniasis	Male	44 years	Negative	A migrant from Brazil; Polar lepromatous leprosy	[14]
Sri Lanka	2010	Extrapulmonary TB	Mucosal leishmaniasis	Male	52 years	Negative	T-lymphocyte subsets: CD3-437, CD4-237, CD8-146 μl; Serum IgG-532 and IgM-36 mg/dl	[15]
India	2010	PTB	Post-kala-azar dermal leishmaniasis	Female	25 years	Positive	On treatment for leprosy and PTB for the past 2 months	[16]
France	2000	Lymph node TB	Lymph node trichomoniasis	Female	82 years	Negative		[17]
India	2010	PTB	Perinatal falciparum malaria	Female	34 days	Unknown	Maternal history of falciparum malaria during eighth month of pregnancy; Father was smear-positive PTB case.	[18]
Haiti	1984	PTB	Toxoplasmosis	Male	27 years	Unknown	Toxoplasmosis proved fatal and was diagnosed only at autopsy.	[19]
Sri Lanka	2011	Lymph node TB	Toxoplasmosis Toxocariasis	Male	4 years	Negative		[20]
Korea	2012	Disseminated TB	Cerebral toxoplasmosis	Male	24 years	Unknown	A non-Korean engineer with abdominal TB	[21]
Australia	2001	PTB Tuberculous lymphadenitis	Hepatosplenic schistosomiasis	Male	30 years		A migrant from the Philippines; A history of alcohol abuse	[22]
India	1991	PTB	Hydatid disease	Female	5 years	Unknown		[23]
Turkey	2002	PTB	Cardiac hydatid cyst	Female	52 years	Negative		[24]
China	2004	TB in liver and abdominal cavity	Liver multiple hydatidosis	Female	28 years	Negative		[25]
Sudan	2009	PTB	Hydatid disease	Female	25 years	Unknown	In a solitary pulmonary nodule of left lower lobe	[26]
China	2009	PTB	Liver echinococcosis	Male	18 years	Unknown	PTB was previously confirmed in 2000 and found relapse in 2008.	[27]
China	2009	PTB	Liver echinococcosis	Female	36 years	Unknown	PTB was previously confirmed in 1998 and found relapse in 2008.	[27]
Britain	1994	PTB	Strongyloidiasis Giardiasis	Male	31 years	Unknown	A migrant from Gambia; Being resident in the United Kingdom for two years	[28]
India	2001	TB verrucosa cutis	Filarial elephantiasis	Male	55 years	Unknown		[29]

Abbreviations: HIV = human immunodeficiency virus; PTB = pulmonary tuberculosis; HCV = hepatitis C virus; RNA = ribonucleic acid; CD = cluster of differentiation; Ig = immunoglobulin.

It can be seen in Table 3 that 5 studies from 3 East African countries and 1 East Asian country were conducted for epidemiology of co-infection between TB and parasitic diseases during 1984 to 2012. Two studies screened across the general population with 382 and 782 participants in a community and a hospital respectively, one study showed 329 and 215 PTB patients in two hospitals respectively, one study showed 309 PTB patients who were HIV positive and 346 who were HIV negative in a hospital, in the other study, there were 112 smear positive TB patients in a town, whose parasite species involved *Entamoeba histolytica*, *Leishmania donovani*, *Giardia lamblia*, malaria, *Clonorchis sinensis*, *Schistosoma mansoni, taenia* species, *Ascaris lumbricoides*, *Trichocephalus trichiurus*, hookworm and *Strongyloides stercoralis*. The prevalence of co-infection between TB and parasitic diseases varied widely among different participants, s species or survey sites.

**Table 3:** Epidemiological surveys of co-infection between TB and parasitic diseases

**Country**	**Year of report**	**No. of population screened (a)**	**No. of people with TB (b)**	**People with parasitic disease (c)**		**No. of people co-infected (d)**	**Prevalence (%)**			**Survey site**	**HIV test**	**No. of reference**
				**No.**	**Species**		**d/a**	**d/b**	**d/c**			
Korea	1984		329		*Trichocephalus trichiurus*	68		20.7		Hospital1	Unknown	[30]
			215		*Trichocephalus trichiurus*	14		6.5		Hospital2	Unknown	
			329		*Clonorchis sinensis*	58		17.6		Hospital1	Unknown	
			215		*Clonorchis sinensis*	13		6.0		Hospital2	Unknown	
Sudan	2004	382	100	252	*Leishmania donovani*	77	20.2	77.0	30.6	Community	Unknown	[31]
Ethiopia	2006	782	100	234	Intestinal parasites	32	4.1	32.0	13.7	Hospital	Unknown	[32]
		782	100	155	Helminths	16	2.0	16.0	10.3	Hospital	Unknown	
					Parasites species							
		782	100	88	*Ascaris lumbricoides*	5	0.6	5.0	5.7	Hospital	Unknown	
		782	100	70	*Giardia lamblia*	15	1.9	15.0	21.4	Hospital	Unknown	
		782	100	50	*Entamoeba histolytica*	10	1.3	10.0	20.0	Hospital	Unknown	
		782	100	36	Hookworm	6	0.8	6.0	16.7	Hospital	Unknown	
		782	100	25	Taenia species	2	0.3	2.0	8.0	Hospital	Unknown	
		782	100	13	*Strongyloides stercoralis*	5	0.6	5.0	38.5	Hospital	Unknown	
		782	100	8	*Trichuris trichiura*	1	0.1	1.0	12.5	Hospital	Unknown	
Tanzania	2007		309		Malaria	19		6.1		Hospital	HIV positive	[33]
			309		Schistosomiasis	100		32.4		Hospital	HIV positive	
			309		Hookworm	33		10.7		Hospital	HIV positive	
			346		Malaria	9		2.6		Hospital	HIV negative	
			346		Schistosomiasis	132		38.2		Hospital	HIV negative	
			346		Hookworm	86		24.9		Hospital	HIV negative	
Ethiopia	2012		112		Intestinal helminths	32		28.6		Community	47% (53/112) of PTB patients were HIV positive	[34]
			112		*Ascaris lumbricoides*	12		10.7		Community		
			112		Hookworm	8		7.1		Community		
			112		*Schistosoma mansoni*	6		5.4		Community		
			112		*Trichuris trichiura*	8		7.1		Community		
			112		*Strongyloides stercoralis*	3		2.7		Community		

The interrelationship of infection with TB and parasitic diseases have been evaluated in Table 4 through two studies screened in the general population, with 382 and 782 participants in a community and a hospital respectively. Those who were *Leishmania donovani* positive, *Giardia lamblia* positive or *Strongyloides stercoralis* positive more easily suffered from PTB than those who were negative and RR values (95% CI) were 1.73 (1.14-2.62), 1.80 (1.10-2.93) and 3.11 (1.53-6.35), respectively. Likewise, persons with PTB were more easily infected by *Leishmania donovani*, *Giardia lamblia* or *Strongyloides stercoralis* than persons without PTB and RR values (95% CI) were 1.24 (1.08-1.43), 1.86 (1.09-3.16) and 4.26 (1.42-12.77), respectively.

**Table 4:** The interrelationship of infection between TB and parasitic diseases

**Country**	**Year of report**	**Parasitic disease**		**PTB (+)**	**PTB (−)**	**RR**_**row **_**(95% CI)**	***P*-value**	**No. of reference**
Sudan	2004	*Leishmania donovani*	+	77	175	1.73 (1.14-2.62)		[31]
			_	23	107	1.00		
		**RR**_**col **_**(95**% **CI)**		1.24 (1.08-1.43)	1.00		0.0067	
Ethiopia	2006	Intestinal parasites	+	32	202	1.10 (0.74-1.63)		[32]
			_	68	480	1.00		
		**RR**_**col **_**(95**% **CI)**		1.08 (0.79-1.47)	1.00		0.6272	
		Helminths	+	16	139	0.77 (0.46-1.28)		
			_	84	543	1.00		
		**RR**_**col **_**(95**% **CI)**		0.78 (0.49-1.26)	1.00		0.3047	
		*Ascaris lumbricoides*	+	5	83	0.42 (0.17-0.99)		
			_	95	599	1.00		
		**RR**_**col **_**(95**% **CI)**		0.41 (0.17-0.99)	1.00		0.0341	
		*Giardia lamblia*	+	15	55	1.80 (1.10-2.93)		
			_	85	627	1.00		
		**RR**_**col **_**(95**% **CI)**		1.86 (1.09-3.16)	1.00		0.0233	
		*Entamoeba histolytica*	+	10	40	1.63 (0.90-2.93)		
			_	90	642	1.00		
		**RR**_**col **_**(95**% **CI)**		1.70 (0.88-3.30)	1.00		0.1145	
		Hookworm	+	6	30	1.32 (0.62-2.81)		
			_	94	652	1.00		
		**RR**_**col **_**(95**% **CI)**		1.36 (0.58-3.19)	1.00		0.4451^*^	
		*Taenia species*	+	2	23	0.62 (0.16-2.36)		
			_	98	659	1.00		
		**RR**_**col **_**(95**% **CI)**		0.59 (0.14-2.48)	1.00		0.7594^*^	
		*Strongyloides stercoralis*	+	5	8	3.11 (1.53-6.35)		
			_	95	674	1.00		
		**RR**_**col **_**(95**% **CI)**		4.26 (1.42-12.77)	1.00		0.0172^*^	
		*Trichuris trichiura*	+	1	7	0.98 (0.15-6.17)		
			_	99	675	1.00		
		**RR**_**col **_**(95**% **CI)**		0.97 (0.12-7.84)	1.00		1.0000^*^	

+: infected; -: noninfected; *: Fisher’s exact test; Abbreviation: PTB = pulmonary tuberculosis; RR = risk ratio; CI = confidence interval.

Immunological research of co-infection between TB and parasitic diseases during 1989 to 2012 are shown in Table 5. In four studies that reported a change of activity of the host’s immune system when the course of TB was aggravated by opisthorchiasis invasion, the activity increased at the acute stage of invasion and decreased at the subacute stage or in the chronization. A study from France showed that co-infecton of American cutaneous leishmaniasis, lepromatous leprosy and PTB downregulated the T-helper (Th) 1 cell response. Four studies on co-infection with *Mycobacterium tuberculosis* (M. TB) and malaria showed that malarial parasites decreased the host’s effective humoral and cellular immune responses to M. TB, and co-infection exacerbated chronic TB, suggesting a competitive antagonist effect between heat shock protein 70 (HSP70) from M. TB and adenosine triphosphate-binding protein (ATPBP) of malaria may exist. Two studies suggested that the impact of co-infection between filarial infection and M. TB infection on the immune response was uncertain. Four studies reported immunomodulation characteristics of co-infection beween TB and intestinal helminths. A study from China indicated that as the echinococcosis chronicity increased, the immune profile in TB patients changed from a Th1 to Th2 response. In three other studies in which the effect of infection of intestinal parasites and *Schistosoma mansoni* on the protective immune response to Bacillus Calmette-Guerin (BCG) vaccination against M. TB and the effect of malaria infection on the effectiveness of novel TB vaccines in protecting against TB were evaluated, the protective efficacy of BCG vaccination was reduced but the effectiveness of novel TB vaccines was unaffected.

**Table 5:** Immunological research of co-infection between TB and parasitic diseases

**Country**	**Year of report**	**Tuberculosis**	**Parasitic disease/species**	**Subject**	**Conclusions**	**No. of reference**
France	2003	PTB	American cutaneous leishmaniasis	A 44-year-old man with triple infection of cutaneous leishmaniasis, lepromatous leprosy, and PTB	Unresponsiveness to IL-12 of patient’s T cells after stimulation with *Leishmania guyanensis*, M*. bovis bacille* Calmette-Guerin, and M*.* leprae antigens suggested the inability to mount an appropriate Th cell response to upregulate the IL-12 receptor expression.	[14]
Gambia	1999	M. TB	Malaria	Review	Malarial parasites can decrease their vertebrate host’s effective humoral and cellular immune responses to M. TB.	[35]
USA	2004	M. TB	*Plasmodium yoelii* NL	Mice	Co-infected mice were less able to contain growth of M. TB in lung, spleen, and liver and had increased mortality.	[36]
Thailand	2006	M. TB	*Plasmodium falciparum*	HSP70 (M. TB) and ATPBP (*Plasmodium falciparum*)	HSP70 and ATPBP share a common molecular function as ATP binding resulting from purine nucleotide binding. Therefore, a competitive antagonist effect between both molecules can be expected.	[37]
Germany	2012	M. TB	*Plasmodium berghei* NK65	An experimental mouse model of co-infection	Co-infection exacerbated chronic TB while rendering mice less refractory to *Plasmodium*. Co-infected animals presented with enhanced inflammatory immune responses as reflected by exacerbated leukocyte infiltrates, tissue pathology and hypercytokinemia accompanied by altered T-cell responses.	[38]
Unknown	1989	PTB	Opisthorchiasis	173 patients with PTB complicated by opisthorchiasis	Among them, activity of the α1-proteinase inhibitor was more frequently higher and carriers of two markers i.e. Hp 2–2 and Gc 1–1 were more frequent.	[39]
Unknown	1992	PTB	Opisthorchiasis	12 PTB patients concurrent with opisthorchiasis	When the course of TB is aggravated by opisthorchiasis invasion, the number of cases of antibacterial therapy intolerance increases and prognosis of the diseases deteriorates. It was shown that the antibacterial and anthelminthic therapy had a favourable clinical and immunologic effect.	[40]
Unknown	1992	M. TB	Opisthorchiasis	Animal	In comparison with the groups of animals with monoinvasion and monoinfection, at the acute invasive phase of mixed pathology (2 weeks) the activity of the host’s immune system increases, while the biological activities of both pathogens decrease; however, at the subacute phase (2.5 months), all were contrary respectively.	[41]
Russia	2003	TB	Opisthorchiasis	Golden hamsters with SRBC parasitocenosis	In cases of mixed pathology, at the acute stage of invasion, the immune system showed increased responses with respect to specific and heterologous antigens; in the chronization of invasion the formation of antibodies to heterologous antigens (SRBC) and the level of specific antiopisthorchiasis and anti-TB immune responses decreased together.	[42]
China	2009	PTB	Echinococcosis	A 18 years old male and a 36 years female	As the echinococcosis chronicity increased, the immune profile in both subjects changed from a Th1 to Th2 response. Such an elevated Th2 immune profile, with subsequent suppression of the Th1 immune response, is a common feature of chronic helminth infections.	[27]
USA	2012	M. TB	*Litomosoides sigmodontis*	Cotton rats with chronic *L. sigmodontis* infection and uninfected controls were challenged with M. TB by intranasal inoculation.	Chronic filarial infections do not exacerbate M. TB infection in the cotton rat model. It may be possible to develop worm-derived therapies for autoimmune diseases that do not substantially increase the risk for infections.	[43]
USA	2012	M. TB	Filarial infections	Review	Filarial infections very clearly alter the magnitude and quality of the mycobacteria-specific cytokine responses, responses that have been typically associated with control of these intracellular pathogens.	[44]
Denmark	2007	TB	Intestinal helminth	Review	Co-infections cause a range of immunomodulation characterized by enhanced Th2-type cytokine profiles, high IgE levels and upregulated regulatory T-cell activity, as well as chronic immune activation.	[45]
Brazil	2007	PTB	Intestinal helminth	40 PTB patients and 25 healthy controls	Compared to either TB patients or healthy controls, co-infected patients’ absolute frequencies of CD3^+^, CD4^+^, CD8^+^, NK T cell and CD4^+^CD25^high^ T cell increased. Differences in CD_4_ T cell frequencies were accompanied by lower IFN-γ and elevated and sustained IL-10 levels in WB cultures from co-infected patients compared to TB patients.	[46]
Mexico	2012	TB	Helminthic infections	Review	Helminths very clearly alter the magnitude of the mycobacteria-specific cytokine responses, altering the control of the mycobacteria growth. Mycobacteria-induced immune responses are suppressed by helminth infections.	[47]
USA	2012	TB	Helminths	Review	Helminth-induced Th2 and T reg responses impinge on host resistance against M. TB infection. Th1 response is reduced in helminth co-infected hosts. Helminth-induced alternatively activated macrophages contribute to enhanced susceptibility to TB.	[48]
Brazil	2002	BCG vaccination	*Ascaris lumbricoides*, *Entamoeba histolytica*, and *Strongyloides stercoralis*	14 male students aged 12–15 years: 7 having protozoan or helminthic infections and 7 free of intestinal parasites	Th2-like IL-10 responses induced by intestinal parasites may interfere in BCG-induced Th1-like IFN-γ response. Intestinal parasitic infections may significantly alter the protective immune response to BCG vaccination.	[49]
Sweden	2005	BCG vaccination to protect against M. TB	*S. mansoni*	BCG vaccinated mice with prior *S. mansoni* infection	*S. mansoni* infection reduces the protective efficacy of BCG vaccination against M. TB possibly by attenuation of protective immune responses to mycobacterial antigens and/or by polarizing the general immune responses to the Th2 profile.	[50]
USA	2011	TB vaccines in protecting against M. TB	*Plasmodium yoelii* NL	TB vaccines in a co-infection mouse model of two pathogens	The effectiveness of novel TB vaccines in protecting against TB was unaffected by a primary malaria co-infection in a mouse model of PTB.	[51]

Abbreviations: PTB = pulmonary tuberculosis; M*.* leprae = *mycobacterium leprae*; M. TB = *mycobacterium tuberculosis*; SRBC = sheep red blood cells; IL = interleukin; Th = T helper; Treg = T regulatory; NL = non-lethal; HSP = heat shock protein; ATP = adenosine triphosphate; ATPBP = ATP-binding protein; Ig = immunoglobulin; CD = cluster of differentiation; NK = natural killer; IFN = interferon; WB = whole blood; BCG = Bacillus Calmette-Guerin; *L. sigmodontis* = *litomosoides sigmodontis*; *S. mansoni* = *schistosoma mansoni*.

## Discussion

This review only found 24 cases of co-infection with TB and parasitic diseases but they distributed widely in 13 countries and covered PTB, extrapulmonary TB, and diseases caused by protozoa and helminths [8-29]. We also found 5 epidemiological studies in which prevalence of co-infection varied widely [30-34], and found that it was evident in 18 studies that the activity of the host’s immune system was altered during co-infection happened [27,34-42,44-51]. Therefore, assessing co-infection characteristics, influencing factors and impact on immunity is important to control and prevention whether for TB or parasitic diseases.

About 17 parasite genera concurrent with M. TB were reviewed as case reports, epidemiological surveys and immunological research, which were divided into protozoa (*Entamoeba*[32,49], *Leishmania*[8-16,31], *Giardia lamblia*[28,32], *Trichomonas*[17], *Plasmodium*[18,33,35,37,38], *Toxoplasma*[19-21]) and helminths (*Clonorchis sinensis*[30], *Opisthorchis*[39,40], *Schistosoma*[22,33,34], *Taenia*[32], *Echinococcus*[23-27], *Ascaris lumbricoides*[32,34,49], *Toxocara*[20], *Trichuris trichiura*[30,32,34], *Ancylostoma*[32-34], *Strongyloides stercoralis*[28,32,34,49], *Filaria*[29,43,44]) and covered most of the common parasites species. Of those, leishmaniasis, hydatid disease and malaria were reported to coexist more freuequently with TB in the human body. In addition to co-infection of TB and a single parasitic disease, there was also co-infection of TB and multiple parasitic diseases, such as co-infection of pneumocystis carinii pneumonia, visceral leishmaniasis and PTB, co-infection of toxoplasmosis, toxocariasis and TB, and co-infection of strongyloidiasis, giardiasis and TB [9,20,28].

Possibly gender differences exist in co-infection of TB and different parasitic diseases. Among ten cases reported to be co-infected with TB and leishmaniasis, only two cases were female [8-16], however, only one of six cases co-infected with TB and hydatid disease was male [23-27]. In spite of these limited data without epidemiological evidence, we can infer that different social roles associated by gender possibly result in different probabilities of contacting different parasites resulting in different incidence and prevalence. An epidemiological survey in subtropical Ecuador suggested that male gender was one of risk factors associated with cutaneous leishmaniasis [53]. Contrarily, a population-based study in the Hamar of Ethiopia indicated that hydatid disease was a public health problem for women [54]. Irrefutably, gender differentials also exist in the prevalence of TB [55].

Although no significant age clustering was found in 23 cases of co-infecion who had a huge age span ranging from 34 days old to 82 years old [8-29], however, there were fewer reported cases of less than 20 years old and more than 60 years old. Likewise, this limited data were not epidemiological results. Nevertheless, age differentials of co-infection also exist and should not be a neglected factor. A cross-sectional household survey in Sudan showed that percentages of leishmania skin tests and tuberculin positivity by age group increased [31], which possibly reflects that with the increasing age, not only does the immune response increase following exposure to infection but also opportunities of exposure to infection increase gradually.

Among TB patients, prevalence of parasitic disease varies widely in different areas and different survey sites. TB and parasitic disease co-infection is common in clinical practice in East Africa. In Sudan, up to 77% of TB patients were positive for the leishmania skin test in the community [31]; 32% of TB patients from hospitals had intestinal parasites and 29% of TB patients from the community had intestinal helminths in Ethiopia [32,34]; and 32.4-38.2% of TB patients from hospitals were also infected with Schistosomes in Tanzania [33]. We also noticed the prevalence differentials in Korea, where the infection rates of *Trichocephalus trichiurus* and *Clonorchis sinensis* were 20.7% and 17.6% respectively among TB patients in one hospital, but the infection rates were 6.5% and 6.0% respectively in another hospital [30]. Among the PTB patients from a hospital in Tanzania, the HIV-positive patients had a significantly lower prevalence of hookworm and Schistosome infection and a higher prevalence of malaria than the HIV-negative patients [33], which indicates HIV also has an impact on prevalence of co-infection.

Therefore, it is no surprise to find many factors that possibly affect co-infection of TB and parasitic diseases. First, socio-demographics, such as gender and age as previously mentioned above, maybe relate to prevalence of co-infection. Second, some special patients, such as renal transplant recipients, patients on maintenance haemodialysis, HIV positive patients and migrants [8,10-12,14,16,21,22,28,33], are likely to be susceptible populations of co-infection. Last but not least, there are higher probabilities of co-infection in some areas with a higher prevalence of TB and parasitic diseases, such as India and East Africa [11,12,16,18,23,29,31-34]. These might provide useful information to control and prevent co-infection of TB and parasites under the background of very few epidemiological surveys for co-infection to date.

It was observed that PTB and parasitic diseases were risk factors for each other. We analyzed the data of an epidemiological survey in Sudan and found that Leishmaniasis patients had a 1.73 times higher risk for PTB than individuals without Leishmania and PTB patients had a 1.24 times higher risk for Leishmaniasis than individuals without PTB [31]. We also found data from an epidemiological survey in Ethiopia that Giardiasis and Strongyloidiasis patients all had higher risks for PTB than persons without *Giardia lamblia* and *Strongyloides stercoralis*, respectively, and PTB patients had a higher risk for Giardiasis and Strongyloidiasis than persons without PTB [32].

In comparison with mono-infection, co-infection makes the host’s immune system have to deal with a more complex internal environment. The usual manifestation is that clinical signs might become more frequent and serious and therapy may be affected when TB patients are also infected by parasites. Both clinical and laboratory based studies showed that when the course of TB was aggravated by opisthorchiasis invasion, clinical signs of TB became more pronounced, disorders in the functions of the liver and pancreas became more frequent, antibacterial therapy intolerance increased and prognosis of the disease deteriorated [39,40]. In order to clarify these situations, some studies were conducted to evaluate the immuno-pathogenesis of co-infection. Limited data from two studies showed that the immune response of TB patients increased at the acute stage of opisthorchiasis invasion and decreased at the subacute stage or in the chronization [41,42]. Two studies indicated that malarial parasites decreased the hosts effective humoral and cellular immune responses to M. TB [35,36], and another study using an experimantal animal model showed co-infection between TB and malaria exacerbated chronic TB while rendering mice less refractory to *Plasmodium*[38]. Another study found that with the increase of echinococcosis chronicity, the immune profile in TB patients displayed an elevated Th2 immune reponse with subsequent suppression of the Th1 immune response [27]. Although one study suggested chronic filarial infections do not exacerbate M. TB infection [43], a review pointed out that filarial infections very clearly alter the magnitude and quality of the mycobacteria-specific cytokine response [44]. Generally, it is observed that the host’s immune system is inhibitited to a great extent during co-infection.

Some investigations observed the change of levels of immune indexes during co-infection. A study on co-infection of PTB and opisthorchiasis found that among co-infected patients, activity of the α1-proteinase inhibitor was more frequently higher and carriers of two markers i.e. Hp 2–2 and Gc 1–1 were more frequent [39]. A study on triple infection of leishmaniasis, TB and leprosy showed that patients were unable to mount a Th cell response to upregulate the interleukin-12 receptor expression after stimulation of the triple infection [14]. The investigation of co-infection between TB and intestinal helminths suggested that compared to either TB patients or healthy controls, the absolute frequencies of CD3^+^, CD4^+^, CD8^+^, Natural killer T cell and CD4^+^CD25^high^ T cell increased in co-infected patients [46]. Indeed, the change of immune index levels reflects a ‘weakened’ immune response.

It is worthwhile evaluating the impact of parasitic disease infection on the efficacy of BCG vaccination against M. TB. Ferreira *et al.*[49] and Elias *et al.*[50] found that intestinal parasitic infections might significantly alter the protective immune response to BCG vaccination and/or polarize the general immune response to the Th2 profile since Th2-like interleukin-10 responses induced by intestinal parasites might interfere in the BCG-induced Th1-like interferon-γ response. Therefore, in areas of high prevalence of co-infection, anti-parasitic chemotherapy prior to immunization may greatly enhance the efficacy of BCG vaccination.

## Conclusion

This review has found limited evidence of factors that influence epidemic and host’s immunity of co-infection between TB and parasitic disease in humans. Most of the common parasites species are concurrent with M. TB in multiple organs, which increase antibacterial therapy intolerance and deteriorate prognosis of disease. Socio-demographics such as gender and age, special populations with susceptibility, such as renal transplant recipients, patient on maintenance haemodialysis, HIV positive patients and migrants, and living in or coming from co-endemic areas likely have impacts on co-infection. PTB and parasitic diseases were shown to be risk factors for each other. Co-infection may inhibit the hosts immune system to a great extent. In addition, infection with parasites can alter the protective immune response to BCG vaccination against M. TB.

## Competing interest

The authors declare that they have no conflicts of interest.

## Authors’ contributions

XZ and XL conceived and designed the review; XL conducted the review of the literature, extracted the pertinent data, performed analysis of data, and wrote the first draft of the manuscript; XZ provided strategic advice and assisted with editing of the manuscript. All authors read and approved the final version of the manuscript.
